# Cyclophilin D Promotes Acute, but Not Chronic, Kidney Injury in a Mouse Model of Aristolochic Acid Toxicity

**DOI:** 10.3390/toxins13100700

**Published:** 2021-10-01

**Authors:** Khai Gene Leong, Elyce Ozols, John Kanellis, Frank Y. Ma, David J. Nikolic-Paterson

**Affiliations:** Monash Medical Centre, Department of Nephrology, Monash Health and Monash University Centre for Inflammatory Diseases, Clayton, VIC 3168, Australia; khaigene.leong@monashhealth.org (K.G.L.); elyce.ozols@monash.edu (E.O.); john.kanellis@monash.edu (J.K.); frank.ma@monash.edu (F.Y.M.)

**Keywords:** acute kidney injury, aristolochic acid, cell death, chronic kidney disease, cyclophilin D, inflammation, renal fibrosis

## Abstract

The plant-derived toxin, aristolochic acid (AA), is the cause of Chinese Herb Nephropathy and Balkan Nephropathy. Ingestion of high dose AA induces acute kidney injury, while chronic low dose ingestion leads to progressive kidney disease. Ingested AA is taken up by tubular epithelial cells of the kidney, leading to DNA damage and cell death. Cyclophilin D (CypD) participates in mitochondrial-dependent cell death, but whether this mechanism operates in acute or chronic AA-induced kidney injury is unknown. We addressed this question by exposing *CypD-/-* and wild type (WT) mice to acute high dose, or chronic low dose, AA. Administration of 5 mg/kg AA to WT mice induced acute kidney injury 3 days later, characterised by loss of kidney function, tubular cell damage and death, and neutrophil infiltration. All of these parameters were significantly reduced in *CypD-/-* mice. Chronic low dose (2 mg/kg AA) administration in WT mice resulted in chronic kidney disease with impaired renal function and renal fibrosis by day 28. However, *CypD-/-* mice were not protected from AA-induced chronic kidney disease. In conclusion, CypD facilitates AA-induced acute kidney damage, but CypD does not contribute to the transition of acute kidney injury to chronic kidney disease during ongoing AA exposure.

## 1. Introduction

Aristolochic acid (AA) is a nitrophenanthrene carboxylic acid found in the *Aristolochiaceae* family that includes almost 500 plants. AA is principally composed of a mixture of two metabolites, the 8-methoxy-6-nitro-phenanthro-(3,4-d)-1,3-dioxolo-5-carboxylic acid (AAI) and 6-nitro-phenanthro-(3,4-d)-1,3-dioxolo-5-carboxylic acid (AAII) [1]. Investigation of Balkan endemic nephropathy identified AA as the nephrotoxin responsible for this environment-associated disease [2]. *Aristolochia* species growing in cereal crop fields in the region contaminated the baking flour, with chronic AA consumption causing chronic kidney disease, nephrolithiasis, and bladder cancer [1]. *Aristolochia* species are also used in the preparation of various Chinese sliming herbs, with AA identified as the causative toxin in Chinese Herb Nephropathy [3]. In one study of 300 cases of Chinese Herb Nephropathy, acute kidney injury and more slowly progressive chronic kidney disease were associated with high or low levels of AA ingestion, respectively [4]. The identification of AA as the common toxin responsible for these diseases has led to the term aristolochic acid nephropathy (AAN). Currently, there is no treatment for AAN and thus understanding the mechanisms by which AA induces acute and chronic kidney injury is critical.

Tubular epithelial cells of the kidney are highly susceptible to AA toxicity since they express the organic anion transporter OAT1/3 which enables efficient uptake of AA into the cell [5]. Within cells, AA reacts with DNA bases, producing DNA adducts that can result in a A:T→T:A transversion which causes DNA damage and can leading to cancer development [1]. In addition, AA induces death in cultured tubular epithelial cells via the induction of high levels of reactive oxygen species (ROS) [6]. Both mice and rats are susceptible to the toxic effects of AA administration, allowing in vivo studies on the mechanism of AA-induced renal toxicity. Acute renal failure with tubular necrosis can be induced in animals by a single high dose of AA, while chronic kidney disease with tubular atrophy and fibrosis can be induced by repeated exposure to low dose AA [7,8].

Cyclophilins are a group of widely expressed enzymes that have peptidyl cis–trans isomerase (PPIase) activity and are involved in protein folding. Cyclophilin D (CypD), also known as Peptidylprolyl Isomerase F (PPIF), is a component of the mitochondrial membrane permeability transition pore (mPTP). Following cell injury, due to excessive ROS or other stressors, the mPTP is opened leading to release of cytochrome c into the cytoplasm and subsequent cell death [9]. Deletion of the *CypD* gene in mice results in a normal phenotype, but *CypD-/-* mice are resistant to tubular necrosis and acute renal failure induced by renal ischaemia/reperfusion injury or cisplatin toxicity [10,11,12,13]. Furthermore, *CypD-/-* mice show protection in the unilateral ureteric obstruction (UUO) model of renal fibrosis [14]. However, it is unknown whether CypD plays a role in AA induced TEC damage. This study investigated the role of CypD in both high dose AA-induced acute kidney injury and in chronic low dose AA-induced renal fibrosis.

## 2. Results

### 2.1. Cyclophilin D Deletion Protects against Aristolochic Acid-Induced Acute Kidney Injury

**Figure 1:**
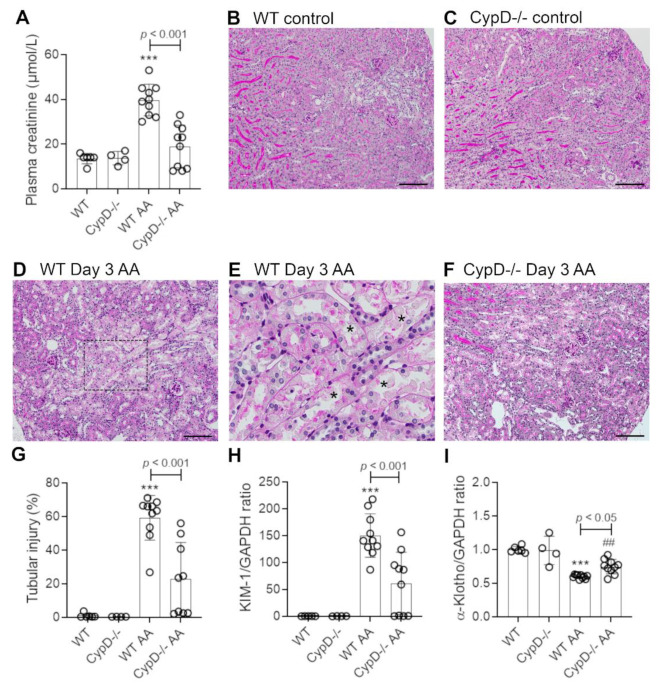
Renal function and tubular damage on day 3 of high dose (5 mg/kg) aristolochic acid (AA)-induced acute kidney injury in wild type (WT) and *CypD-/-* mice, compared to control mice without experimentation. (**A**) Plasma creatinine levels. (**B**–**F**) Periodic acid-Schiff (PAS) staining of kidney sections. Normal kidney structure with tightly packed tubular cells with clear brush border staining in (**B**) WT control, and (**C**) *CypD-/-* control kidney. (**D**) WT on day 3 after AA showing damaged tubules with loss of brush border, loss of tubular nuclei and sloughing of cells into the lumen. (**E**) High power view of the dashed area in (**D**), with examples of necrotic tubules indicated by asterisks. (**F**) *CypD-/-* on day 3 after AA shows reduced tubular damage compared to WT day 3 AA mice. Bars = 200 μm. (**G**) Score of tubular damage. (**H**,**I**) Reverse transcription PCR (RT-PCR) analysis of mRNA levels for; (**H**) Kim1, and; (**I**) α-Klotho. One-way ANOVA with Tukey’s multiple comparison test; *** *p <* 0.001 vs. WT control, ^##^ *p <* 0.01 vs. *CypD-/-* control.

**Figure 2:**
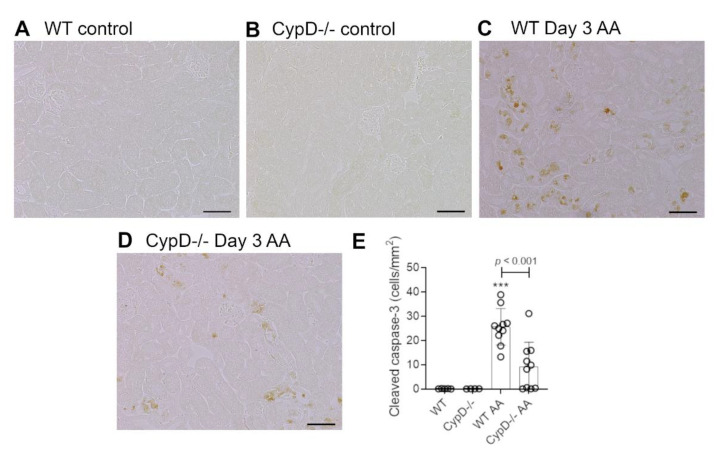
Tubular cell death on day 3 of high dose (5 mg/kg) aristolochic acid (AA)-induced acute kidney injury in wild type (WT) and *CypD-/-* mice, compared to control mice without experimentation. (**A**–**D**) Immunostaining for cleaved caspase 3. No staining is seen in (**A**) WT control, or (**B**) *CypD-/-* control kidney. (**C**) WT on day 3 after AA showing numerous tubular epithelial cells with nuclear and some cytoplasmic staining for cleaved caspase 3. (**D**) *CypD-/-* on day 3 after AA shows fewer tubular cells stained for cleaved caspase 3. Bars = 100 μm. (**E**) Graph quantifying the number of cleaved caspase 3 stained cells. One-way ANOVA with Tukey’s multiple comparison test; *** *p <* 0.001 vs. WT control.

Healthy wild type (WT) C57BL6/J mice have plasma creatinine levels in the range of 8 to 16 μmol/L. Administration of 5 mg/kg AA to WT mice induced an acute reduction in kidney function on day 3 as defined by a 3-fold increase in plasma creatinine levels (range of 30 to 53 μmol/L) (Figure 1A). Compared to WT control mice (Figure 1B), significant histologic damage to tubular epithelial cells was evident in WT mice on day 3 after AA administration involving; loss of the apical brush border, cell swelling, loss of cell nuclei, sloughing of cells into the tubular lumen, and cast formation in the tubular lumen (Figure 1D). Tubular necrosis is clearly seen in the high-power view in Figure 1E. We also evaluated cell death by staining for cleaved caspase 3. WT control mice lack staining for cleaved caspase 3, but numerous tubular epithelial cells showed cleaved caspase 3 staining on day 3 after AA administration (Figure 2A,C). Consistent with these histological features of cell damage and cell death, there was a marked increase in the mRNA levels for the tubular damage marker Kim1, and a significant reduction in the mRNA level of the protective protein, α-Klotho (Figure 1H,I).

Mice with global *CypD* gene deletion (*CypD-/-*) have normal kidney structure and function (Figure 1A,C). *CypD-/-* mice were substantially protected from acute kidney injury induced by a single administration of 5 mg/kg AA. While 5/10 *CypD-/-* mice showed a mild increase in plasma creatinine levels, the average plasma creatinine level was substantially lower than the WT AA group, and was not significantly different to *CypD-/-* without AA treatment (Figure 1A). A similar picture was evident in the analysis of tubular damage, with the *CypD-/-* day 3 AA group showing significant reductions in histologic tubular injury, the number of cleaved caspase-3 stained cells, and Kim1 mRNA levels (Figure 1F–H and Figure 2D,E), and significant protection against a reduction in α-Klotho mRNA levels (Figure 1I).

### 2.2. Cyclophilin D Deletion Protects against Acute Aristolochic Acid-Induced Leukocyte Infiltration

**Figure 3:**
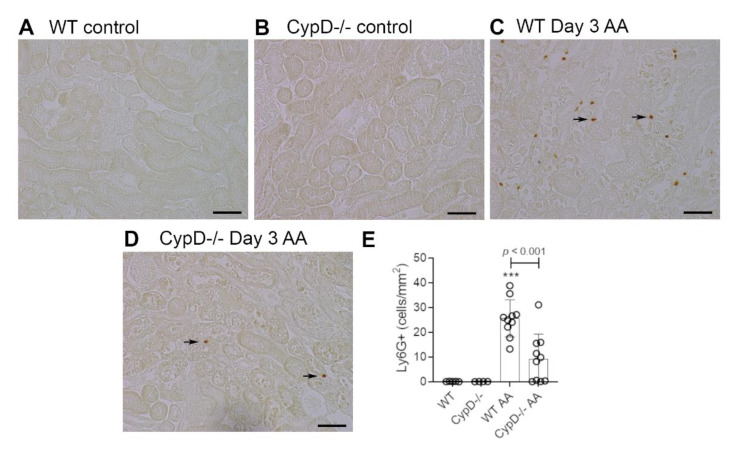
Neutrophil infiltration on day 3 of high dose (5 mg/kg) aristolochic acid (AA)-induced acute kidney injury in wild type (WT) and *CypD-/-* mice, compared to control mice without experimentation. (**A**–**D**) Immunostaining for Ly6G+ neutrophils. No neutrophils are seen in (**A**) WT control, or (**B**) *CypD-/-* control kidney. (**C**) WT on day 3 after AA showing infiltrating neutrophils around damaged tubules (arrows indicate examples of neutrophil staining). (**D**) *CypD-/-* on day 3 after AA shows a reduction in neutrophil infiltration. Bars = 100 μm. (**E**) Graph quantifying the number of Ly6G+ neutrophils. One-way ANOVA with Tukey’s multiple comparison test; *** *p <* 0.001 vs. WT control.

**Figure 4:**
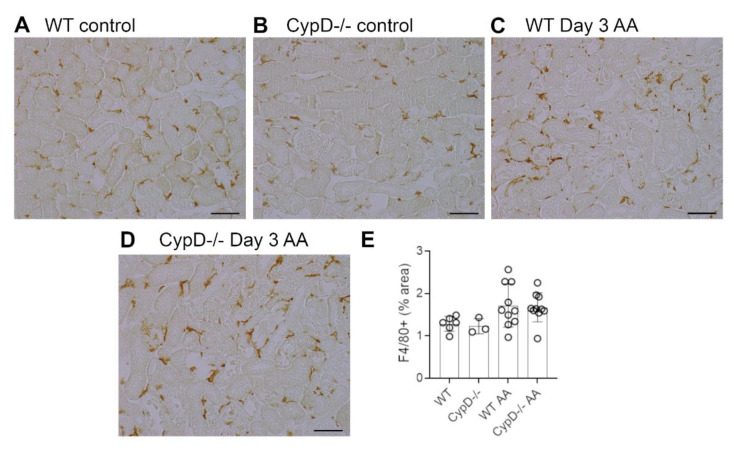
Macrophage infiltration on day 3 of high dose (5 mg/kg) aristolochic acid (AA)-induced acute kidney injury in wild type (WT) and *CypD-/-* mice, compared to control mice without experimentation. (**A**–**D**) Immunostaining for F4/80+ macrophages. A network of F4/80+ resident macrophages is evident in (**A**) WT control, and (**B**) *CypD-/-* control kidney. (**C**) WT on day 3 after AA shows no overall increase in macrophage numbers. (**D**) *CypD-/-* on day 3 after AA also shows no change in macrophage numbers. Bars = 100 μm. (**E**) Graph quantifying the area of F4/80 staining.

**Figure 5:**
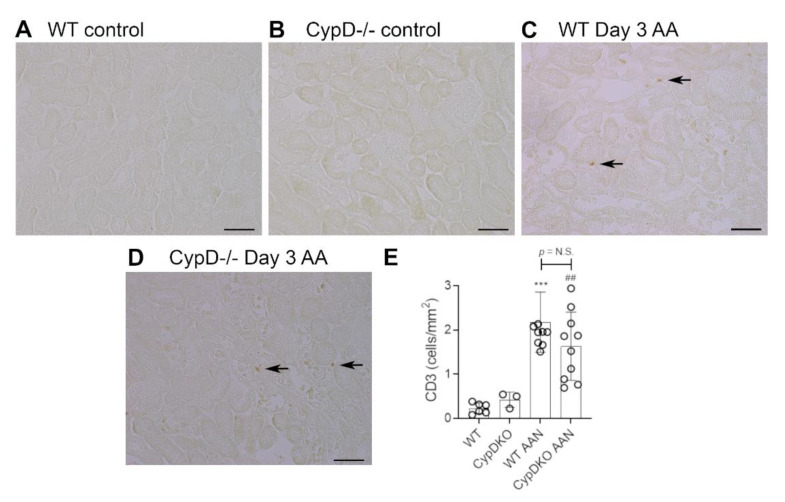
T cell infiltration on day 3 of high dose (5 mg/kg) aristolochic acid (AA)-induced acute kidney injury in wild type (WT) and *CypD-/-* mice, compared to control mice without experimentation. (**A**–**D**) Immunostaining for CD3+ T cells. Most areas of the kidney cortex lack CD3+ T cells in (**A**) WT control, and (**B**) *CypD-/-* control mice. (**C**) WT on day 3 after AA shows a small number of infiltrating T cell (examples shown by arrows). (**D**) *CypD-/-* on day 3 after AA show a similar small number of infiltrating T cells. Bars = 100 μm. (**E**) Graph quantifying the number of CD3+ T cells. One-way ANOVA with Tukey’s multiple comparison test; *** *p <* 0.001 vs. WT control, ^##^ *p <* 0.01 vs. *CypD-/-* control. N.S., not significant.

Neutrophils are largely absent in WT and *CypD-/-* control kidneys (Figure 3A,B). However, a substantial infiltrate of Ly6G+ neutrophils was evident in areas of tubular damage in WT mice on day 3 after AA administration (Figure 3C). This neutrophil infiltrate was substantially reduced in the *CypD-/-* AA group (Figure 3D,E). In contrast to neutrophils, there is a substantial population of resident F4/80+ macrophages in WT and *CypD-/-* control kidneys (Figure 4A,B). While there was some increase in macrophages in areas of tubular damage, this did not reach statistical significance in either the WT AA or *CypD-/-* AA groups (Figure 4C–E). T cells are largely absent in WT and *CypD-/-* control kidneys (Figure 5A,B). A small, but significant, T cell infiltrate was seen on day 3 AA in both WT and *CypD-/-* kidneys, although this was not different between WT and *CypD-/-* mice (Figure 5C–E).

### 2.3. Cyclophilin D Deletion Does Not Protect against Chronic Aristolochic Acid-Induced Kidney Disease

**Figure 6:**
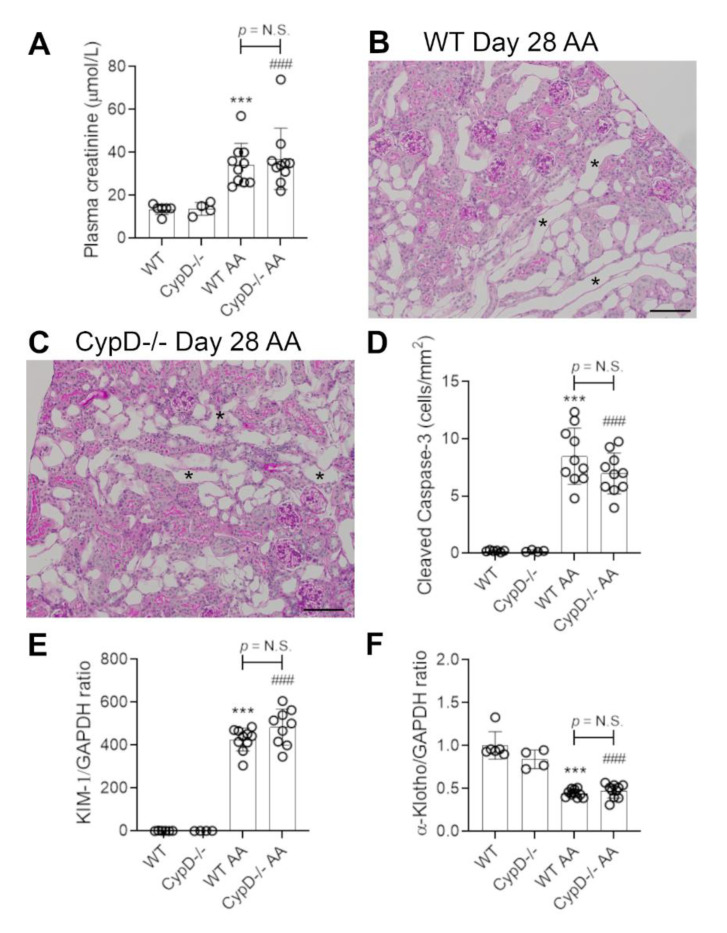
Renal function and tubular damage on day 28 of repeated low dose (2 mg/kg) aristolochic acid (AA) administration in wild type (WT) and *CypD-/-* mice, compared to control mice without experimentation. (**A**) Plasma creatinine levels. (**B**) PAS staining of WT on day 28 showing extensive tubular atrophy and interstitial cell infiltration. Examples of atrophic tubules are shown by asterisks. (**C**) PAS staining of *CypD-/-* on day 28 also shows extensive tubular atrophy and interstitial cell infiltration. Bars = 150 μm. (**D**) Graph quantifying cleaved caspase 3 stained cells. (**E**,**F**) Reverse transcription PCR (RT-PCR) analysis of mRNA levels for; (**E**) Kim1, and; (**F**) α-Klotho. One-way ANOVA with Tukey’s multiple comparison test; *** *p <* 0.001 vs. WT control, ^###^ *p <* 0.001 vs. *CypD-/-* control. N.S., not significant.

Administration of 2 mg/kg AA to WT mice every second day, for a period of 28 days resulted in chronic kidney disease, with a 2.6-fold increase in plasma creatinine levels (Figure 6A). Histologic examination revealed prominent tubular atrophy and dilatation, some tubular cast formation and cellular infiltration in the interstitium, although glomeruli remained largely normal (Figure 6B). No obvious tubular necrosis was evident in WT day 28 AA (Figure 6B), but a significant number of cleaved caspase 3 stained cells (presumably apoptotic cells) were evident (Figure 6D), although this was reduced by 66% compare to day 3 WT AA (Figure 2E; *p* < 0.001). Kim1 mRNA levels were increased, and α-Klotho mRNA levels decreased in the day 28 WT AA group (Figure 6E,F); both of these changes were of greater magnitude than that seen in the day 3 WT AA group (Figure 1H,I; both *p* < 0.001).

*CypD-/-* mice were not protected from kidney disease induced by chronic AA administration. They exhibited plasma creatinine levels, histologic damage, Kim1 and α-Klotho mRNA levels, and numbers of cleaved caspase 3 stained cells comparable to that seen in day 28 AA WT mice (Figure 6A,C–F).

### 2.4. Cyclophilin D Deletion Does Not Protect against Chronic Aristolochic Acid-Induced Renal Fibrosis

**Figure 7:**
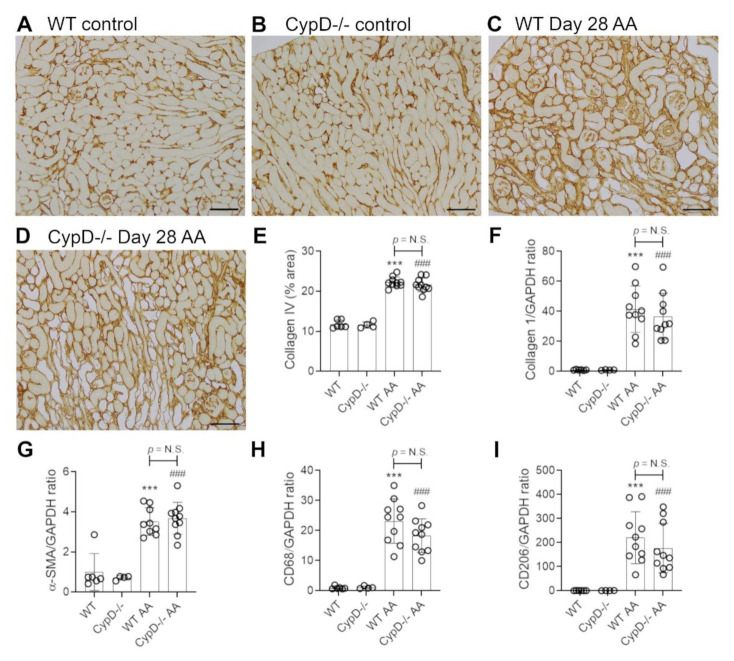
Renal fibrosis on day 28 of repeated low dose (2 mg/kg) aristolochic acid (AA) administration in wild type (WT) and CypD-/- mice, compared to control mice without experimentation. (**A**–**D**) Immunostaining for collagen IV. Collagen IV staining of glomerular and tubular basement membranes is seen in (**A**) WT control, and (**B**) *CypD-/-* control kidney. (**C**) WT day 28 AA shows a diffuse increase in collagen IV staining in the interstitial area. (**D**) *CypD-/-* day 28 AA also shows increased interstitial collagen IV staining. Bars = 200 μm. (**E**) Graph quantifying the area of collagen IV staining. (**F**–**I**) Reverse transcription PCR (RT-PCR) analysis of mRNA levels for; (**F**) collagen I; (**G**) α-SMA; (**H**) CD68, and (**I**) CD206. One-way ANOVA with Tukey’s multiple comparison test; *** *p <* 0.001 vs. WT control, ^###^ *p <* 0.001 vs. *CypD-/-* control. N.S., not significant.

**Figure 8:**
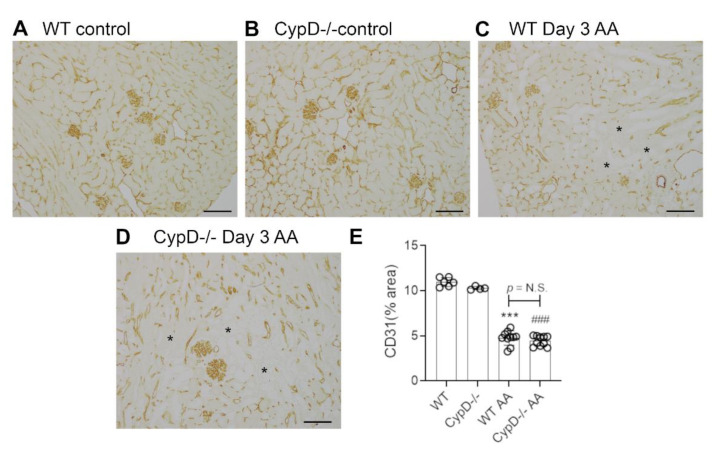
Loss of peritubular capillaries on day 28 of repeated low dose (2 mg/kg) aristolochic acid (AA) administration in wild type (WT) and *CypD-/-* mice, compared to control mice without experimentation. (**A**–**D**) Immunostaining for CD31. Staining of CD31+ endothelial cells in the peritubular and glomerular compartments is seen in (**A**) WT control, and (**B**) *CypD-/-* control kidney. (**C**) WT day 28 shows a partial loss of interstitial CD31+ endothelial cells. Asterisks show areas with loss of peritubular capillaries. (**D**) *CypD-/-* on day 28 also shows a partial loss of interstitial CD31+ endothelial cells. Bars = 200 μm. (**E**) Graph quantifying the area of CD31 staining. One-way ANOVA with Tukey’s multiple comparison test; *** *p <* 0.001 vs. WT control, ^###^ *p <* 0.001 vs. *CypD-/-* control. N.S., not significant.

Renal interstitial fibrosis is a hallmark of chronic kidney disease which features collagen deposition, accumulation of α-SMA+ myofibroblasts, macrophage infiltration and loss of peritubular capillaries [15]. Compared to WT and *CypD-/-* control kidneys in which collagen IV is localised to glomerular and tubular basement membranes (and blood vessel walls) (Figure 7A,B), day 28 AA WT mice exhibited a diffuse increased in collagen IV deposition in the interstitial area (Figure 7C). This 2-fold increase in interstitial collagen IV deposition (Figure 7E), was associated with a significant increase in kidney collagen I mRNA levels (Figure 7F). A significant myofibroblast accumulation was indicated by a 3.5-fold increase in α-SMA mRNA levels (Figure 7G). A significant macrophage infiltrate was also evident on day 28 AA in WT mice as shown by increased CD68 mRNA levels (Figure 7H), with a dramatic increase in the expression of CD206 (Figure 7I)—a marker of alternatively activated macrophages that has been implicated in promoting renal fibrosis [15]. In addition, day 28 AA WT mice showed a significant loss of CD31+ peritubular capillaries compared to WT control mice (Figure 8A,C,E).

Day 28 AA *CypD-/-* mice were not protected from renal fibrosis, with no appreciable difference compared to day 28 AA WT mice in terms of collagen IV deposition (Figure 7D,E), increased mRNA levels for collagen I, α-SMA, CD68 or CD206 (Figure 7F–I), or the loss of CD31+ peritubular capillaries (Figure 8B,D,E).

## 3. Discussion

Tubular necrosis is a feature of acute kidney injury in situations where toxic chemicals, such as medications (e.g., cisplatin, vancomycin, gentamycin and colistin) and plant nephrotoxins (e.g., aristolochic acid and atractyloside), are preferentially taken up by kidney tubular epithelial cells [16]. A common feature of this toxin-induced tubular cell death is damage to mitochondria. For example, in addition to causing DNA damage, high dose cisplatin or AA-induced acute kidney injury is directly linked to severe mitochondrial damage in kidney tubular epithelial cells [17,18].

CypD plays an important role in toxin-induced, mitochondrial-dependent cell death via the opening of the mPTP [9]. This study demonstrates, for the first time, that CypD is required for high dose AA-induced tubular epithelial cell death and acute kidney injury. This conclusion is based on *CypD-/-* mice showing significantly better renal function, reduced histologic tubular injury and tubular cell death denoted by cleaved caspase 3 staining, reduced expression of the tubular damage marker Kim-1, and protection against loss of α-Klotho expression. These findings are consistent with previous in vitro studies showing that AA induces tubular epithelial cell death via the induction of mitochondrial-derived reactive oxygen species [19], and that CypD is required for reactive oxygen species induced cell death in cultured tubular epithelial cells [14]. In addition, *CypD-/-* mice showed a significant reduction in neutrophil accumulation in the acute AA model. Relatively little is known about the function of CypD in neutrophils; however, this reduction in neutrophil infiltration may simply be an indirect effect of less tubular cell damage and cell death, where reduced release of danger-associated molecular patterns results in reduced neutrophil recruitment. While neutrophils contribute to a “second wave” of tubular cell death in models of renal ischaemia/reperfusion injury [20,21,22], the role of neutrophils per se in acute AA-induced acute kidney injury has not been determined. A minor, but significant infiltrate of T cells was also evident in day 3 AA, but this was not different between WT and *CypD-/-* mice. No difference in kidney macrophage numbers were evident in the day 3 AA model compared to controls.

The demonstration that *CypD-/-* mice are protected from high dose AA-induced acute kidney injury is consistent with studies showing that *CypD-/-* mice are protected from tubular epithelial cell death and loss of kidney function following cisplatin administration or renal ischaemia/reperfusion injury (IRI) [10,11,12], and that treatment with a cyclophilin inhibitor can protect against IRI-induced acute kidney injury [23].

Having established that *CypD-/-* mice are protected from high dose AA-induced acute kidney injury, we hypothesized that these mice would also be protected from tubular cell death and progressive kidney disease induced by chronic exposure to low dose AA. However, this was not the case, with *CypD-/-* mice exhibiting tubular cell damage (based on Kim-1 and α-Klotho), renal failure and renal fibrosis at levels indistinguishable from WT mice in response to chronic administration of low dose AA.

There are several possible reasons for the lack of protection of *CypD-/-* mice during chronic exposure to low dose AA. Histological analysis shows tubular necrosis in the acute, high dose AA model, but tubular necrosis is not evident on day 28 of the chronic low dose AA model when damage is evident in terms of atrophy and apoptotic cell death (shown by staining for cleaved caspase 3). This argues that high dose AA, but not low dose AA, induces tubular necrosis. This might be explained by differences in the induction of reactive oxygen species induced by low versus high dose AA, or that surviving tubular cells develop resistance to AA-induced necrosis. Further detailed studies would be required to separate these possible mechanisms—or identify another potential explanation such as tubular cell damage in the chronic model of AA exposure resulting from accumulated DNA damage. However, it is clear that *CypD* gene deletion cannot protect tubules against the repeated insult of low dose AA administration. Indeed, a similar finding was seen using an inhibitor of the JUN amino terminal kinase (JNK) in these acute and chronic AA exposure models [24]. Both JNK and CypD are involved in ROS-induced, mitochondrial-dependent tubular cell death [14,24]. While JNK inhibitor treatment suppressed tubular cell death in the acute high dose AA model, it had no impact upon tubular cell death or the development of renal fibrosis in the chronic low dose AA model [24].

The specific role of CypD in renal fibrosis has been described in three previous studies. In the unilateral ureteric obstruction (UUO) model, systemic delivery of a cyclophilin inhibitor or the use of *CypD-/-* mice showed protection against tubular cell death, myofibroblast accumulation, collagen deposition and loss of peritubular capillaries [14,23]. However, cultured WT and *CypD-/-* kidney fibroblasts showed no difference in PDGF-induced cell proliferation or TGF-β1 induced activation and collagen production [14], indicating that CypD does not directly affect the collagen producing myofibroblasts in the development of renal fibrosis. Thus, the protection of *CypD-/-* mice against renal fibrosis in the UUO model was attributed to reducing death of tubular epithelial cells and the loss of peritubular capillaries [14]. By contrast, another study found that *CypD-/-* mice exhibit worse glomerulosclerosis in a model of streptozotocin-induced type 1 diabetic kidney disease, while treatment with a cyclophilin inhibitor failed to modify the development of glomerulosclerosis in the *db/db* model of type 2 diabetic kidney disease [25]. The current study found no effect of *CypD* deficiency in the development of renal fibrosis in the chronic low dose AA exposure model, with no effect upon death of tubular epithelial cells or the loss of peritubular capillaries. These contrasting findings in three different disease models point to the role of CypD in the development of renal fibrosis as being highly dependent on the nature of the underlying renal insult and may not be of general importance to renal fibrosis.

## 4. Conclusions

This study demonstrates that CypD contributes to acute tubular necrosis and acute kidney injury induced by exposure to high dose AA. By contrast, CypD does not promote the toxic effects of chronic low dose AA exposure in the induction of chronic kidney disease.

## 5. Materials and Methods

### 5.1. Materials

Aristolochic Acid I (A9451, sodium salt) was obtained from Sigma-Aldrich (Castle Hill, NSW, Australia). Primary antibodies utilised in these experiments were: rat anti-mouse Ly6G to detect neutrophils (Abcam, Melbourne, Victoria, Australia); rat anti-mouse F4/80 to detect macrophages (Bio-Rad, Gladesville, NSW, Australia); rabbit anti-CD31 to detect endothelial cells (Cell Signaling, San Diego, CA, USA); rabbit anti-cleaved caspase 3 to detect cell death (Cell Signaling), and; goat anti-collagen IV (Southern Biotechnology, Birmingham, AL, USA). Biotinylated secondary antibodies (rabbit anti-goat IgG, rabbit anti-rat IgG, or goat anti-rabbit IgG) and the VECTASTAIN Elite ABC HRP Kit for immunoperoxidase staining were obtained from Vector Laboratories (Burlingame, CA, USA).

### 5.2. Mice

*CypD-/-* (also known as *Ppif-/-*) mice on the C57BL6/J background were obtained from JAX Mice and Services (Bar Harbor, ME, USA) and bred at the Monash Animal Research Precinct (MARP), Australia. Wild type C57BL/6J control mice were obtained from MARP. All animal experimentation was approved by the Monash Medical Centre Animal Ethics Committee (MMCB/2017/06), and was performed in accordance with 8th Edition of the Australian National Health and Medical Research Council guidelines for animal experimentation.

### 5.3. Aristolochic Acid-Induced Acute and Chronic Kidney Injury

Acute kidney injury was induced in groups (n = 10) of 10- to 12-week-old male wild type (WT) and *CypD-/-* mice by a single intraperitoneal injection of high dose (5 mg/kg) AA dissolved in saline. Chronic kidney disease was induced in groups (n = 10) of 10- to 12-week-old male wild type (WT) and *CypD-/-* mice by intraperitoneal injections of low dose (2 mg/kg) AA dissolved in saline given every second day from day 0 until being killed on day 28. Groups of 4 to 6 mice without experimentation were used as controls for each genotype.

### 5.4. Histology

Renal histology was assessed in sections (2 μm) of formalin-fixed tissue stained with periodic acid-Schiff (PAS) and hematoxylin. Tubular cell morphology in the day 3 AA model was assessed across the entire cortex under high power (X400). Damage tubules were defined as exhibiting one or more of the following features: tubular dilation or atrophy, loss of brush border, loss of tubular nuclei, and cast formation. Scoring was performed on blinded slides.

### 5.5. Immunostaining

Immunoperoxidase staining for Ly6G+ neutrophils, F4/80+ macrophages and collagen IV was performed on 4 μm sections of paraffin-embedded, methylcarn-fixed kidney slices as previously described [14]. After deparaffinisation and rehydration, the sections were washed in PBS (3 × 5 min) and blocked with 5% bovine serum albumin (BSA) in PBS for 30 min. Sections were then incubated overnight at 4 °C in primary antibody diluted in 1% BSA in PBS. After washing in PBS, endogenous peroxidase activity was blocked using 3% hydrogen peroxidase in distilled water, followed by incubation with the Avidin and then Biotin blocking reagent for 15 min each according to the manufacturer’s instructions. Then, sections were incubated with biotinylated secondary antibody in 1% BSA/5% normal mouse serum in PBS for 60 min, washed, incubated with the avidin-biotin-peroxidase complex for 30 min, washed and the peroxidase enzyme detected with 3,3′-diaminobenzidine. Sections were then dehydrated in graded ethanol, soaked in histolene, and mounted.

Immunoperoxidase staining for cleaved caspase 3 and CD31 was performed on paraffin sections (4 μm) of formalin-fixed kidney. After deparaffinisation and rehydration, antigen retrieval was performed 0.1 mol/L sodium citrate pH 6.0 at 95 °C using a Decloaking Chamber NxGen (Biocare Medical, Concord, CA, USA). Sections were then stained using the 3-layer peroxidase method described above.

The number of cells stained for cleaved caspase 3, Ly6G or CD3 were counted in ×250 power fields covering the entire cortex and expressed as cells per mm^2^. The area of interstitial F4/80, collagen IV and CD31 staining was analysed in ×400 power fields (large blood vessels and glomeruli were excluded from the analysis) covering the entire cortex using image analysis with cellSens software version 1.18 (Olympus Australia, Notting Hill, Victoria, Australia). All analyses were performed on blinded slides.

### 5.6. Real Time Polymerase Chain Reaction

Total cellular RNA extraction from frozen kidney tissue, cDNA synthesis, and PCR reactions on a StepOne Real-Time PCR System (Applied Biosystems, Foster City, CA, USA) were performed as previously described [14]. Taqman primer/probes were purchased from Applied Biosystems. The comparative Ct (ΔCt) method was used to quantify the relative amount of mRNA which was normalized against the internal *Gapdh* mRNA control.

### 5.7. Statistical Analysis

Data are shown as mean ± SD. Analysis used one-way ANOVA with Tukey’s multiple comparison test and was performed using GraphPad Prism 9.0 (San Diego, CA, USA).

## Data Availability

Data is available upon reasonable request.
